# Laryngopharyngeal Reflux Is a Potential Risk Factor for Juvenile-Onset Recurrent Respiratory Papillomatosis

**DOI:** 10.1155/2019/1463896

**Published:** 2019-02-10

**Authors:** Martin Formánek, Pavel Komínek, Debora Jančatová, Lucia Staníková, Radoslava Tomanová, Jana Vaculová, Milan Urík, Ivo Šlapák, Karol Zeleník

**Affiliations:** ^1^Department of Otorhinolaryngology and Head and Neck Surgery, University Hospital Ostrava, 17. Listopadu 1790, 70852 Ostrava, Czech Republic; ^2^Department of Pathology, University Hospital Ostrava, 17. Listopadu 1790, 70852 Ostrava, Czech Republic; ^3^Faculty of Medicine, Masaryk University, Kamenice 5, 625 00 Brno, Czech Republic

## Abstract

**Introduction:**

Human papillomavirus (HPV) causes juvenile-onset recurrent respiratory papillomatosis (JORRP). Although HPV is common in children, the prevalence of JORRP is low. It is likely that other factors contribute to the pathogenesis of JORRP, during either activation or reactivation of a latent HPV infection. There is evidence that laryngopharyngeal reflux (LPR) might be such a risk factor for adult-onset recurrent respiratory papillomatosis. This study investigated if LPR might also be a risk factor for JORRP.

**Materials and Methods:**

Children with JORRP of the larynx that required microlaryngoscopy at a tertiary referral hospital were included in this prospective case-series study from November 2015 to November 2017. Using immunohistochemistry, HPV infection and pepsin associated with LPR were diagnosed from laryngeal biopsies.

**Results:**

Eleven children (aged 4-14 years) were analyzed. No patient had a history of immunodeficiency or tobacco smoke exposure. All patients underwent at least three previous surgeries due to JORRP and had been vaccinated against HPV in the past. Five children were treated using antivirotics and immunomodulators. The only known maternal risk factor was that three mothers were primiparous. All 11 samples were infected with HPV (type 6 or 11). Pathologic LPR was diagnosed in 5/11 children (45.5%).

**Conclusion:**

LPR may be a risk factor for JORRP, contributing to its development by activating or reactivating a latent HPV infection. Results are in accordance with those from our previous study in adults.

## 1. Introduction

Juvenile-onset recurrent respiratory papillomatosis (JORRP) is a chronic viral disease affecting children. It is characterized by the growth of squamous cell tumors in the mucosa of the aerodigestive tract, with a predilection for the larynx. JORRP is the most common benign neoplasm of the larynx among children and the second most frequent cause of childhood hoarseness. Its incidence is 0.17-1.34 and prevalence 1.69-3.88 per 100,000 children. These values are higher in Africa than in Europe, North America, or Australia [1–6] and no difference in prevalence was found between the sexes [7]. In addition to hoarseness, the most common symptoms are stridor and respiratory distress [8]. JORRP is potentially very aggressive and tends to recur. Although it is benign, it has an unpredictable clinical course, can spread into the respiratory tract, and can undergo malignant conversion [9, 10].

JORRP is caused by human papillomavirus (HPV) infection. It is a sexually transmitted disease, and children are mostly infected from HPV-positive mothers during vaginal delivery [11]. The risk of transmission is elevated among young primiparous mothers with condylomas [12]. Despite the low incidence of JORRP, the presence of asymptomatic HPV is relatively common in the respiratory tracts of children [13, 14]. Therefore, other factors have to contribute to the pathogenesis of JORRP, during either the activation or reactivation of HPV. It is unclear how HPV infection progresses to JORRP. The roles of local laryngeal irritants (i.e., tobacco, reflux, and secondhand smoke) in the acquisition, progression, and aggressiveness of disease are controversial. Some data question the dogma that active and passive smoking plays a role in recidivistic disease [15–17]. Several authors have also questioned the role of laryngopharyngeal reflux (LPR), mainly because of the lack of well-designed studies and less than ideal diagnostic methods of LPR, which were mostly limited to questionnaires or indirect signs of reflux, in existing studies [16, 18, 19].

In our recent study in adults, LPR was significantly more frequent in patients with adult-onset recurrent respiratory papillomatosis (AORRP) than control patients with healthy laryngeal mucosa, indicating LPR might be a risk factor for AORRP [15]. This study aimed to investigate if LPR might also be risk factor for JORRP using the same diagnostic scheme.

## 2. Materials and Methods

This prospective case-series study was approved by the Ethics Committee under identifier: 315/2014. It was performed in accordance with the Declaration of Helsinki, with good clinical practice, and it followed the applicable regulatory requirements. The study was registered at ClincialTrials.gov under the identifier: NCT02592902. Written informed consent was obtained from the parents before initiating any procedure. The study was conducted from November 2015 to November 2017 at a tertiary referral hospital.

Children who had been diagnosed with JORRP of the larynx were included in the study if they had histologically confirmed, recurrent (at least two times during the previous two years) squamous cell papillomas in the laryngeal mucosa. Exclusion criteria were patients with contraindications for general anesthesia; patients with laryngeal papillomas undergoing microlaryngoscopy for the first time; and patients whose parents did not consent to participation in the study. Altogether there were 14 eligible patients. Two patients did not meet the inclusion criteria and one patient was excluded because his/her parents did not consent to participation in the study.

Biopsy specimens of laryngeal papillomas were obtained during microlaryngoscopy procedures. Paraffin-embedded sections (2–3 *μ*m thick) were prepared from the biopsy samples (Benchmark XT, Roche, Ventana Medical Systems, Switzerland) and analyzed at the Department of Pathology by a single pathologist. In preparation for immunohistochemistry, samples were treated with hydrogen peroxide to block endogenous peroxidase (Roche) and with Cell Conditioning 1 buffer (Roche) for antigen revitalization. Papillomavirus Mouse Monoclonal Antibody (types 6, 11, and 18) (NCL-HPV-4C4, Leica Biosystems, Germany, concentration 1:5) was used as the primary antibody to detect HPV. Anti-pepsin antibody (NB100-66518, Novus Biologicals, USA, concentration 1:100) was used as the primary antibody to detect pepsin. Samples were incubated with primary antibody for 32 min. The results were visualized with the iView DAB Detection Kit (Roche). The presence of any antibody positivity in the cytoplasm of the cells was considered pathological and the sample was identified as pepsin positive.

The following variables were examined: the child's age, tobacco smoke exposure, history of immunodeficiency, HPV vaccination and previous treatment, the number of previous JORRP surgeries, whether the mother was primiparous, the age of the mother at delivery, the mother's history of condylomata, the results of Papanicolaou test in the period around childbirth, the presence and type of HPV, and the presence of pepsin in the biopsy. Statistical analysis was performed using Microsoft Excel.

## 3. Results

This study included eleven children with JORRP, six girls and five boys. The average age was 8 years. No patient had a history of immunodeficiency or tobacco smoke exposure. All patients had voice problems and coughs, while no patient suffered from heartburn or dysphagia. All patients underwent at least three previous surgeries due to JORRP. All patients had been vaccinated against HPV (Silgard®) in the past. Five children were treated by antivirotics and immunomodulators. The only known maternal risk factor was that three mothers were primiparous (Table 1). Histological findings confirmed squamous cell papillomas in all samples. There was no malignity. Immunohistochemical analyses showed that HPV had infected all 11 samples; HPV type 6 was confirmed in nine samples and HPV type 11 in two samples. Pepsin was detected in five samples, indicating the presence of relevant pathologic LPR (Table 1).

## 4. Discussion

JORRP is a chronic viral disease caused by HPV. Currently, over 120 types of HPV have been identified. They are grouped according to pathophysiology and tissue preference [8, 20]. JORRP is mostly caused by HPV types 6 and 11, which are the etiological agents of over 90% of genital warts [8, 21]. In addition, specific viral subtypes may be correlated with disease severity and the clinical course; for example, an HPV type 11 infection is associated with a poor course and a poor prognosis [22]. In the present study, HPV (type 6 and 11) was confirmed in all biopsy specimens in children with JORRP, despite the fact that all children were vaccinated against HPV with three doses of Silgard® vaccine (0, 2, and 4 month intervals) before the beginning of our study. This finding is consistent with the vaccine not being therapeutic, as all children were vaccinated after the onset of JORRP.

Although the risk of HPV transmission is higher for children with young, primiparous mothers with condylomas, no mother in our case series had a history of condylomata and three mothers were primiparous. In addition, no mother reported a pathological Papanicolaou test in the period around childbirth. However, we did not have the exact results; therefore we had to rely only on anamnestic data that may not be accurate. In contrast to the relatively low incidence of JORRP, the reported prevalence of HPV is relatively high. Szydlowski et al. [13] reported that HPV was present in the respiratory tract in 19.6% of healthy preschool children, while Rintala et al. [14] reported that 10% of healthy children had HPV in the respiratory tract at a 3-year follow up after birth. Durzynska et al. found that HPV was present in the oropharynx in 1.08% of asymptomatic children aged 10-18 years. The most frequent HPV genotypes were types 6 and 11 [23]. Those findings suggested that additional risk factors might contribute to the development of JORRP by activating or reactivating a latent HPV infection.

It was previously shown that LPR could cause or facilitate many pathologies in children, including acute or chronic otitis media and laryngitis [24, 25]. Many studies demonstrated that contact between refluxed material and the mucosa caused local inflammation and edema. The presence of pepsin in the refluxate is considered a primary pathogenic factor, which causes proteolysis and cell damage. LPR could potentially induce JORRP by a very similar mechanism. Evidence has shown that gastroesophageal reflux occurs in approximately two-thirds of infants at 4 months of age, but decreases as the child matures [26–28]. All children in our study were 4 years and older; therefore, LPR should not be present in these patients. The diagnosis of LPR is challenging. Questionnaires are not suitable and the symptoms of LPR are both heterogeneous and very common. The situation is even more complicated in case of JORRP. Voice problems are very often symptoms of LPR, but they are always present in JORRP. Moreover, JORRP can cause other symptoms related to reflux, like globus pharyngeus and cough. Symptoms of JORRP were the dominant complaints in our study. As expected, no children suffered from heartburn as it is only pathognomonic for gastroesophageal reflux.

Therefore, it is very important to choose the proper diagnostic method for evaluating whether LPR is a relevant factor. Measuring pepsin in fluids or tissues appears to be the most suitable method for evaluating a possible relationship between a pathology and LPR [24, 29]. The advantage of pepsin detection over esophageal impedance is that pepsin can be detected in tissues and fluids, even in the absence of a reflux event within the past several days [24]. Moreover, the presence of pepsin inside the mucosal cell cytoplasm indicates that LPR is a relevant factor, because it is typically absent in the cytoplasm of healthy laryngeal mucosa. Thus, this is one of the benefits of this test compared with the more often used lavage of the laryngeal/tracheal mucosa, in which pepsin is assayed in the aspirate and only intraluminal pepsin is tested. An intraluminal presence does not necessary mean that the pepsin overcame the cell's protecting mechanism and LPR is a relevant factor. In our case, intraluminal pepsin could also provide false positive results, considering the nonphysiological conditions during intubation and general anesthesia, in which there is significant risk of aspiration [30]. Therefore, the pepsin measurement used in our study provided the most accurate data. The disadvantage is that the technique is invasive. However, that was not an issue as the papillomas had to be removed. In our study, pepsin was detected in the cell cytoplasm in 5 of 11 children with JORRP, regardless of the HPV type. Accordingly, these patients suffered from relevant pathologic LPR affecting the larynx. This finding indicated that LPR could be a risk factor for JORRP in these patients and may affect its course.

In accordance with previously reported data, passive smoking was not a factor in our study. No child was exposed to tobacco in his or her family. In addition, no child had a history of immunodeficiency. Thus, these general risk factors did not affect the results. Our observations suggested that the LPR could be a potential risk factor for JORPP, contributing to its development by activating or reactivating a latent HPV infection. To our knowledge, this is the first report to demonstrate a possible relationship.

A limit of our study is the small sample size. However, the aim was to evaluate another risk factor of JORRP and to encourage the initiation of other studies. Another limitation was the absence of a control group, which would be clearly beneficial. However, any biopsy of children's healthy laryngeal mucosa or any LPR diagnostic procedure (e.g., esophageal impedance) in healthy children would be unethical. Children with vocal cysts could be included in the control group. Healthy mucosa would be obtained from reduction of the mucosa flap after cyst removal, as was done previously in a study of adults [15]. However, this pathology is very rare in children and such a small biopsy specimen is not always suitable for analysis. We believe children with other glottic or subglottic pathologies would not be suitable for inclusion in the control group because of a risk of bias due to LPR being a potential etiopathogenetic factor of such pathologies. Thus, healthy mucosa distant from the pathology would have to be removed and this would not be approved. However, no pepsin should be present in the cell cytoplasm of healthy laryngeal mucosa. Therefore, any pepsin positivity shows relevant LPR affecting the examined tissue. This was also confirmed in our previous study where no adults in the control group with healthy laryngeal mucosa were pepsin positive [15]. Case-control studies are needed to confirm these preliminary results in children. It remains unclear how and whether LPR should be treated in cases of JORRP. It is unknown if treatment of LPR alters the course of the disease, as it could also play a role only in its onset. Further studies addressing this issue are needed.

## 5. Conclusions

LPR may be a risk factor for JORRP by activating or reactivating a latent HPV infection. The findings here are in accordance with results of our previous study in adults.

## Figures and Tables

**Table 1:** Characteristics of the study participants.

Patient	1	2	3	4	5	6	7	8	9	10	11
Sex	F	M	F	M	F	F	F	M	M	F	M

Age (years)	4	6	8	11	8	6	4	8	14	9	10

No. of previous JORRP surgeries	5	7	13	11	5	3	7	11	4	4	5

Primiparous mother	yes	no	no	no	no	no	yes	yes	no	no	no

Age of mother at delivery (years)	22	27	27	24	30	29	23	22	28	25	26

Mother with history of condylomata	no	no	no	no	no	no	no	no	no	no	no

Inosine pranobex	no	no	no	yes	no	yes	no	yes	yes	yes	no

HPV type	11	6	6	6	6	6	6	6	6	11	6

Pepsin	pos.	pos.	neg.	neg.	pos.	neg.	pos.	neg.	neg.	neg.	pos.

F = female, M = male, JORRP = juvenile-onset recurrent respiratory papillomatosis, pos. = positive, and neg. = negative.

## Data Availability

All anonymized clinical and histopathological data that have been analyzed are in Table 1. The samples of the patients which were analyzed are stored in Department of Pathology, University Hospital Ostrava (address: 17 Listopadu 1790, 708 52 Ostrava, Czech Republic).
